# An Iterative Fast Microphone Array Design Method Employing Equilateral Triangular Subarrays

**DOI:** 10.3390/s26051696

**Published:** 2026-03-07

**Authors:** Xiaobin Hong, Wentao Yao, Yuanming Chen, Ruimou Cai

**Affiliations:** 1 School of Mechanical & Automotive Engineering, South China University of Technology, Guangzhou 510641, China; scut_hongxiaobin@126.com (X.H.); 202320100884@mail.scut.edu.cn (W.Y.); 2 School of Civil Engineering & Transportation, South China University of Technology, Guangzhou 510641, China; 3 Guangzhou Shipyard International Company Limited, Guangzhou 511462, China; cairm123@126.com

**Keywords:** microphone array design, acoustic imaging, acoustic beamforming

## Abstract

In industrial acoustic imaging, microphone array design is often limited by the strong frequency dependence of array performance, the high computational cost of optimization, and the expense of deploying large numbers of microphones. Most existing optimization-based methods require simultaneous optimization of all array elements, resulting in long design times and limited flexibility in controlling element count. To overcome these limitations, this paper proposes a fast and iterative microphone array design method using equilateral triangular subarrays as basic units. Instead of optimizing the entire array at once, the proposed method incrementally adds subarrays, and in each iteration, a genetic algorithm optimizes only the placement of the newly added subarray for a specified target frequency. By exploiting the rotational symmetry of the equilateral triangular subarrays and the geometric characteristics of the array point spread function, the number of optimization variables and the computational domain are significantly reduced, enabling efficient array design. The proposed method allows frequency-specific performance optimization while providing direct control over the number of array elements, achieving a practical balance between imaging performance and hardware cost. Comparative results show that arrays designed using this method generally exhibit improved main lobe width and sidelobe level performance near the target frequencies compared with several classical array configurations.

## 1. Introduction

Acoustic imaging technology utilizes a phased microphone array as hardware, combined with software algorithms to locate and quantify sound sources in space. It has been widely applied in scenarios requiring sound source localization, such as aircraft wing wind tunnel tests and the detection of honking vehicles on highways [1]. For instance, Michel et al. [2] utilized microphone arrays to measure aerodynamic noise during commercial aircraft landings and identified the noise concentration areas of moving vehicles on roads [3]; Licitra et al. [4] employed this technique to detect leakage points in yacht cabins for enhanced onboard comfort; Petrica [5] successfully detected the sound of illegal logging in forests using acoustic imaging technology; and Moradshahi et al. [6] improved the accuracy of cough detection and sound source localization through microphone arrays.

Many application cases show that the design characteristics of a microphone array directly dictate the quality of acoustic imaging across different frequencies. A well-designed array should have the highest possible resolution and the ability to resist spurious source contamination, ensuring that the acoustic imaging results are clear and readable. However, despite decades of development in acoustic imaging, no universally optimal array design has established, and microphone array performance is always constrained by frequency range; performance degrades to varying degrees when operating beyond the suitable frequency band. Consequently, in factory workshop applications of acoustic imaging, such as separately detecting fans and transmission gears with different characteristic frequencies, acoustic imaging is challenged by the inability of a single microphone array configuration to satisfy diverse detection needs. Furthermore, achieving superior performance typically necessitates a large number of microphones, which significantly increases hardware costs and the complexity of data acquisition systems. Therefore, there is an urgent need for a method that can rapidly optimize array configurations for specific frequency characteristics while minimizing the number of elements to reduce costs. Various optimization methods have been introduced into microphone array design to enhance array performance. For instance, Yu and Iman Khatami et al. [7,8,9,10,11,12,13] explored genetic algorithms, while Malgoezar et al. [14] employed differential evolution algorithms to optimize array configurations for clearer imaging in target areas. Arcondoulis and Liu [15] incorporated topology optimization techniques from structural mechanics into array configuration design. Sarradj [16] proposed a parametric array optimization approach that does not rely on numerical optimization. Son [17] explored the use of simulated annealing to derive a general sparse microphone array suitable for various beam patterns. Most existing optimization strategies focus on adjusting the configuration of predefined array schemes, making it difficult to effectively reduce the total number of elements during the optimization process. These methods are also often computationally complex and time-consuming, rendering them impractical for industrial scenarios requiring frequent directional optimization. Although some studies have adopted an iterative “subtractive” approach to remove redundant elements [15], being able to reduce the total number of elements, such methods typically require a massive initial grid, and the process of identifying and eliminating redundancies remains computationally expensive. Additionally, some researchers have also attempted stochastic and exhaustive search algorithms, but these methods require extensive computational time and are impractical for scenarios requiring multiple targeted optimizations [18].

To overcome these limitations, this paper aims to propose an iterative design method based on an “additive” philosophy. Unlike methods that rely on subtractive filtering from a predefined large-scale grid, this method builds the array from scratch by progressively adding elements. We employ a genetic algorithm (GA) as the optimization tool and introduce equilateral triangular subarrays, each consisting of three elements, as the basic iterative units. The combination of equilateral triangular subarrays and genetic algorithms proves to be a superior strategy: first, the triangular layout effectively balances element spacing to avoid severe spatial aliasing caused by highly regular spacing; second, leveraging the rotational symmetry of the subarrays significantly reduces the number of optimization variables in the GA, thereby enabling fast design. Results from comparisons with classical arrays indicate that the arrays designed by the proposed method typically achieve performance advantages near the target frequencies; combined with its rapid design capability, this provides a feasible microphone array optimization scheme for acoustic imaging tasks in factory workshops detecting various equipment.

The remainder of this paper is organized as follows: Section 2 introduces the beamforming algorithm and array performance metrics; Section 3 details the iterative design method and the implementation of the genetic algorithm; Section 4 presents the experimental results and discussion; Section 5 suggests future research directions; and Section 6 concludes the study.

## 2. Beamforming and Array Evaluation

**Figure 1:**
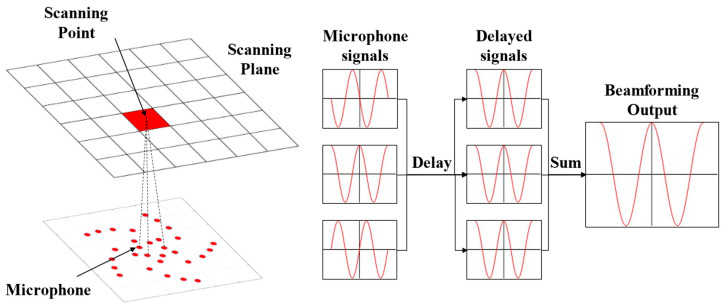
Acoustic imaging using delay-and-sum beamforming.

The hardware foundation of acoustic imaging technology is the phased microphone array, whereas its software counterpart is the Beamforming algorithm. The simplest and most fundamental beamforming method is the delay-and-sum beamforming (DAS), also referred to as conventional beamforming, known for its efficiency and straightforward implementation. The core concept behind this method is to introduce precise time delays to the signals from different channels of the microphone array, aligning them at a specific point in the scanning plane. According to the principle of constructive interference, the point exhibiting the highest signal intensity during scanning corresponds to the actual sound source location [19,20,21], as shown in Figure 1. Nevertheless, the DAS beamforming algorithm is prone to spatial aliasing due to the inherent periodicity of the signal. Beyond time-domain processing, the DAS algorithm can also be adapted to the frequency domain for analyzing signals at designated frequencies.

In practical implementation, beamforming algorithms typically preset a gridded scanning plane. The DAS result at the grid point is obtained by calculating the distances from the grid point to each microphone element, applying corresponding time delays to all channels, and then performing average summation. At a specific point *X* on the grid, the DAS beamforming output B(*X*, *t*) of an array consisting of *N* microphones can be expressed as

(1) $$B\left(X,t\right)=\frac{1}{N}\overset{N}{\underset{n=1}{\sum }}W_{n}\cdot P_{n}\left(t-\Delta t_{n,X}\right)$$

Here *P_n_* represents the time-series sound pressure signal received by the *n*-th microphone element. *W_n_* represents the weight of the *n*-th microphone signal, which typically accounts for the attenuation of sound intensity with distance. And Δ*t_n,X_* represents the time delay of the *n*-th microphone sound signal propagating from point *X* to the microphone. In the frequency domain, the output of DAS is given by

(2) $$B\left(X,\omega \right)=\frac{1}{N}\overset{N}{\underset{n=1}{\sum }}W_{n}\cdot P_{n}\left(\omega \right)e^{j\omega \Delta t_{n,X}}$$

*P_n_*(*ω*) represents the Fourier transform of the *n*-th microphone signal for the frequency *ω*.

In most applications of acoustic source imaging technology, complex sound sources can be approximated as multiple monopole sources [22]. The performance of an array is typically evaluated using its response to a unit-strength monopole source, known as the Point Spread Function (PSF). However, studies indicate that when beamforming is applied to a localize near-field spherical wave source, the performance evaluation of microphone arrays loses its general applicability [23]. Therefore, the PSF generated by a unit-strength far-field plane wave source is used for analysis. Assuming in Equation (2) that the point sound source becomes a plane wave source that has an intensity of 1 and propagation attenuation is ignored (*W_n_* = 1, *P_n_* = 1), the microphone array PSF output Y(***k***, ω) with the coordinates represented in vector form can be written as

(3) $$Y\left(k,\omega \right)=\frac{1}{N}\overset{N}{\underset{n=1}{\sum }}e^{j\cdot \frac{\omega }{c}\cdot \left(k-k_{0}\right)\cdot x_{n}}$$

Here ***k*** represents the unit direction vector from the geometric center *O* of the microphone array plane to the scanning point *X*. It can also be interpreted as the unit vector of the array focus direction. ***k*_0_** represents the direction vector of the unit-strength far-field plane wave, ***x_n_*** denotes the position vector of the *n*-th microphone in the microphone array, *ω* is the frequency of the acoustic signal, and *c* is the speed of sound propagation. Noted that *Y* depends only on (***k*** − ***k*_0_**) and is independent of the absolute value of ***k*_0_**, the shape of *Y* is unaffected by the direction of the incoming wave. Therefore, the computation of *Y* can be simplified by considering only the case where the far-field plane wave is perpendicularly incident on the array plane as ***k*_0_** = (0, 0, −1).

**Figure 2:**
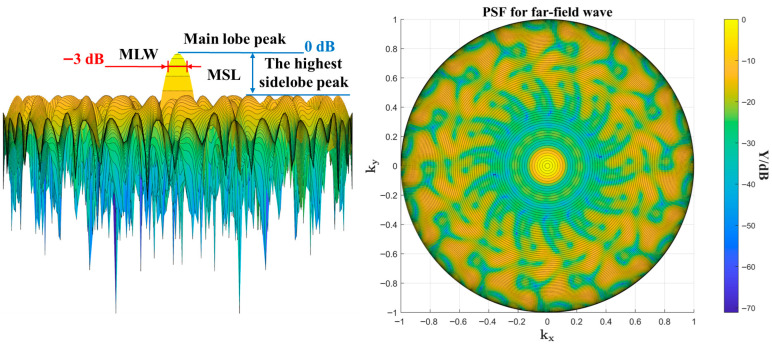
Example of the array point spread function (PSF), where MLW denotes the width around the main lobe at the −3 dB points, and MSL represents the difference between the highest sidelobe level and the main lobe peak.

The image of the PSF output *Y* is shown in Figure 2. The array’s peak response to the incoming wave is normalized to 0 dB, and the width of the −3 dB region around this peak is termed the main lobe width (MLW). The difference between the highest sidelobe peak and the main lobe peak is termed the maximum sidelobe level (MSL). A smaller MLW indicates higher spatial resolution and more accurate source localization, and a lower MSL indicates weaker interference from sidelobes, making it easier to avoid artifacts in acoustic imaging. In this paper, MLW and MSL are used as performance metrics to compare existing well-established classical array configurations with the arrays designed by the proposed method.

## 3. Array Design Method

Numerous existing studies have demonstrated the feasibility and reliability of using genetic algorithms to search for optimal array configurations [7,8,9,10,11,12,13], and mature genetic algorithm toolboxes can substantially reduce the computational complexity of array optimization design. On the other hand, research [24] shows that dividing the array elements evenly and optimizing only part of them, with the rest determined via rotational and symmetric operations, can effectively reduce computational complexity without obvious performance loss compared with optimizing all elements simultaneously. Therefore, combining the genetic algorithm with this region-partition-based design method is clearly a worthwhile direction for further study.

**Figure 3:**
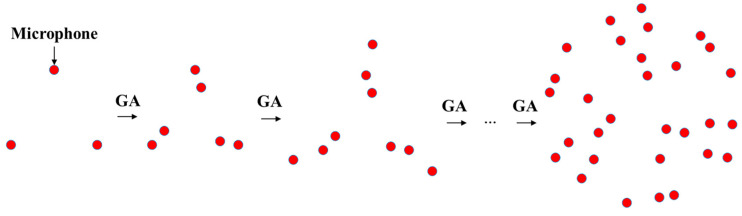
Iterative microphone array design method employing equilateral triangular subarrays with the position of each added subarray optimized via a genetic algorithm.

**Figure 4:**
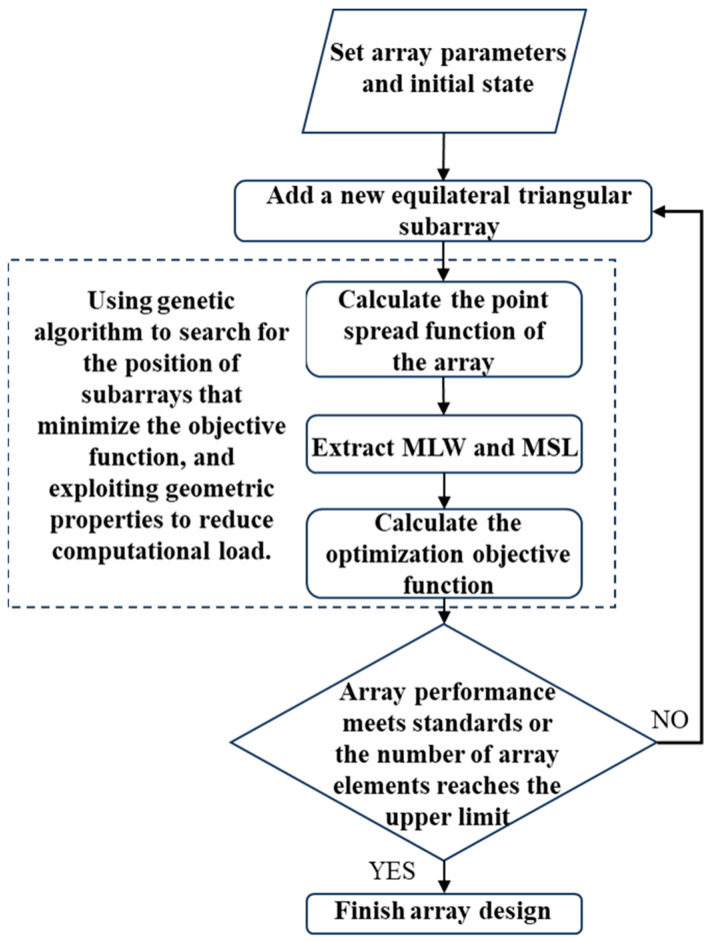
The specific iterative calculation process of the method proposed by this paper.

This paper proposes an iterative microphone array design method that uses three elements arranged in an equilateral triangle as the basic unit. In each iteration, a new equilateral triangular subarray is added to the previous iteration’s result. The process continues until the desired array performance is achieved or a maximum number of elements is reached, with the position of each added subarray optimized via a genetic algorithm. The geometric center of each equilateral triangular subarray is aligned to the array center, resulting in rotational symmetry of the three elements about the center. Rather than optimizing all elements simultaneously, the iterative design reduces the number of optimization variables to only the coordinate parameters of the subarray added in each iteration. And the rotational symmetry among the three elements within each subarray can be exploited to reduce the number of optimization parameters to the coordinates of a single element, thereby significantly reducing the complexity of the design method. Equilateral triangular subarrays as basic units are also selected in consideration of the array element spacing. To avoid spatial aliasing while using a limited number of microphones in a finite-sized array, it is generally believed that the microphone spacing within the array should be irregular, and in contrast, highly regular microphone spacing can lead to severe spatial aliasing [25]. Each equilateral triangular subarray introduces three equal inter-element distances (corresponding to the triangle’s three equal sides), while regular polygons with four or more sides generate four or more such equal distances, which can degrade array performance. The specific iterative calculation process of the method proposed is illustrated in Figure 3 and Figure 4.

In computing the PSF to extract MLW and MSL, the array focus direction unit vector ***k*** can be expressed using azimuth angle *φ* and elevation angle *θ*. Due to the equilateral triangular subarrays, the array has 120° rotational symmetry around its center in the azimuthal plane; and to simplify computation, only the case of a normally incident far-field plane wave, i.e., ***k*_0_** = (0, 0, −1), is considered. Under these conditions, *Y* also exhibits rotational symmetry about the *Z*-axis passing through the array center, with a 120° azimuthal period, which can be written as

(4) $$Y\left(\theta ,\varphi ,\omega \right)=Y\left(\theta +120{}^{\circ},\varphi ,\omega \right)$$

Therefore, only one-third of the full azimuth angular domain of *Y* needs to be evaluated to obtain MLW and MSL, effectively minimizing computation and accelerating processing. More precisely, the full computation domain of *Y* for angular frequency ω is given by

(5) $$Y\left(\theta ,\varphi ,\omega \right),\;0\leq \theta \leq 360^{\circ },\;0\leq \varphi \leq 90^{\circ }$$

which can be simplified as

(6) $$Y\left(\theta ,\varphi ,\omega \right),0\leq \theta \leq 120^{\circ },0\leq \varphi \leq 90^{\circ }$$

After computing *Y*, the MSL is defined as

(7) $$\mathrm{MSL}=20\log_{10}\left(Y_{s}\right)-20\log_{10}\left(Y_{0}\right)=20\log_{10}\left(\frac{Y_{s}}{Y_{0}}\right)$$

where *Y_S_* denotes the unnormalized maximum sidelobe level, *Y*_0_ denotes the unnormalized main lobe level. Since the −3 dB region of the main lobe in *Y* is not a standard circular area, the MLW is searched along four directions to reduce computation time, specifically

(8) $$\mathrm{MLW}=2sin^{-1}\left(max\left\{L_{0},L_{30},L_{60},L_{90}\right\}\right)$$

where *max*{·} represents the maximum value of the elements in curly brackets, *L*_0_ denotes the distance from the −3 dB point to the *Z*-axis in the azimuthal direction of 0°, and *L*_30_, *L*_60_, and *L*_90_ are defined similarly.

The Genetic Algorithm (GA) is an optimization search method rooted in natural selection and genetic principles, drawing inspiration from biological evolution. Based on population search and enhanced by stochastic and heuristic mechanisms, GA offers strong global optimization capabilities in complex nonlinear problems [8]. Thus, it is employed in the proposed array design method to identify the optimal positions of equilateral triangular subarrays that maximize array performance. As the microphone array performance is evaluated using MLW and MSL, the genetic algorithm’s objective function is constructed from both parameters, each normalized into dimensionless form to prevent either from dominating the optimization. The objective function is defined as

(9) $$V=\frac{\mathrm{MLW}}{W}\cdot \frac{Y_{s}}{Y_{0}}$$

where *W* represents the theoretical main lobe width [26], which is given by

(10) $$W=\sin^{-1}\left(\frac{\lambda }{D}\right)$$

where *λ* denotes the signal wavelength, and *D* denotes the array aperture.

## 4. Results and Discussion

In the following experiments, the iterative array design is restricted to 30 elements and an aperture not exceeding 1 m, corresponding to an element radial range from 0 to 0.5 m. In each iteration, the first subarray is moderately characterized by one of its elements with a radial distance of 0.25 m and an azimuth angle of 0°. And the inter-element spacing is constrained to no less than 0.02 m to avoid physical interference between microphones during practical installation. In the experiments, the genetic algorithm was realized using the *ga* function from the Global Optimization Toolbox in MATLAB R2022b, with selected parameters provided in Table 1 and all remaining unspecified parameters set to the default values. The experiments were conducted on a computer powered by a 13th Gen Intel^®^ Core™ i5-13500HX CPU (Intel Corporation, Santa Clara, CA, USA) featuring a base clock speed of 2.50 GHz, 14 cores, and 20 logical processors. Experimental results indicate that the proposed iterative method requires roughly 26 min to design a 30-element array, compared to over 2 h for directly optimizing all 30 elements via a genetic algorithm with the same objective function, demonstrating a clear time advantage.

**Figure 5:**
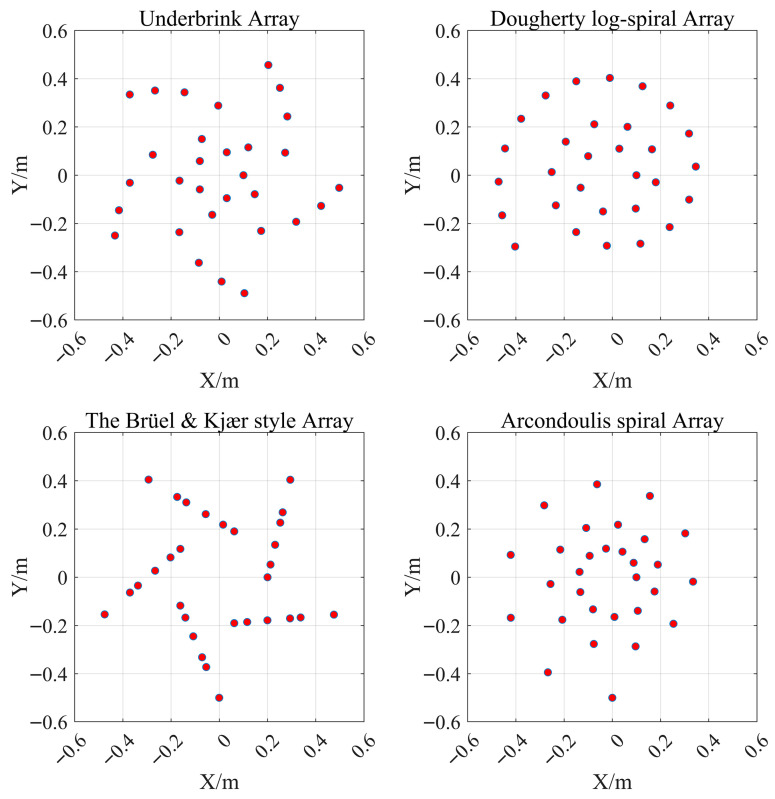
The control group arrays configurations. The red dots represent the microphones.

To confirm the practical performance improvement of the arrays designed using the method proposed in this paper, we implemented several well-known arrays with a 1 m aperture and 30 elements, following array design formulas from References [23,27], as the control group arrays, including Underbrink array, Dougherty log-spiral array, Brüel & Kjær (B&K) style array, and Arcondoulis spiral array as shown in Figure 5.

**Figure 6:**
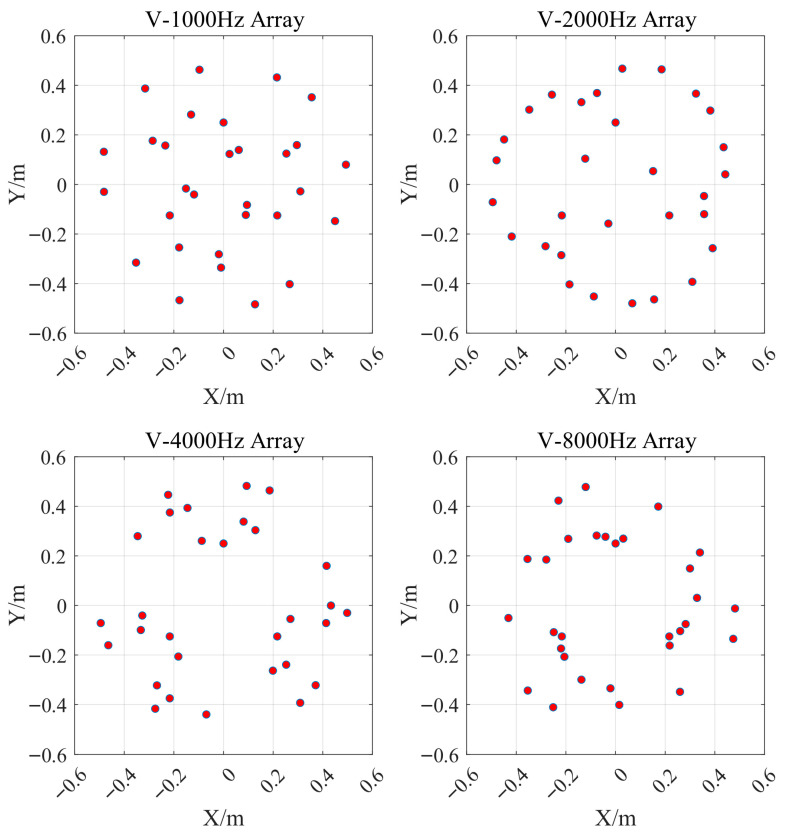
V arrays configurations designed for four frequencies using the method proposed in this paper. The red dots represent the microphones.

Several 30-element arrays were designed by the proposed method to be evaluated against the control group arrays at the target frequency points. The target frequency points were chosen as 1000 Hz, 2000 Hz, 4000 Hz, and 8000 Hz, allowing clear observation of array performance variations with frequency, while the doubling relationship between frequencies further highlights the trend. Also, according to Equation (10), the theoretical MLW becomes excessively large at very low frequencies when *D* is limited. When the frequency is too high, *λ* becomes much smaller than the inter-element spacing *d*, violating the Nyquist requirement *d* < *λ*/2 and leading to stronger sidelobe interference, i.e., increased MSL. In practice, array design must consider a finite aperture *D*, and the inter-element spacing cannot be too small to avoid installation interference, so extreme frequency conditions are unsuitable for assessing array configurations performance. Therefore, this paper analyzes the array performance at the four frequencies mentioned above. The arrays designed by the proposed method are termed the V arrays, as illustrated in Figure 6.

**Figure 7:**
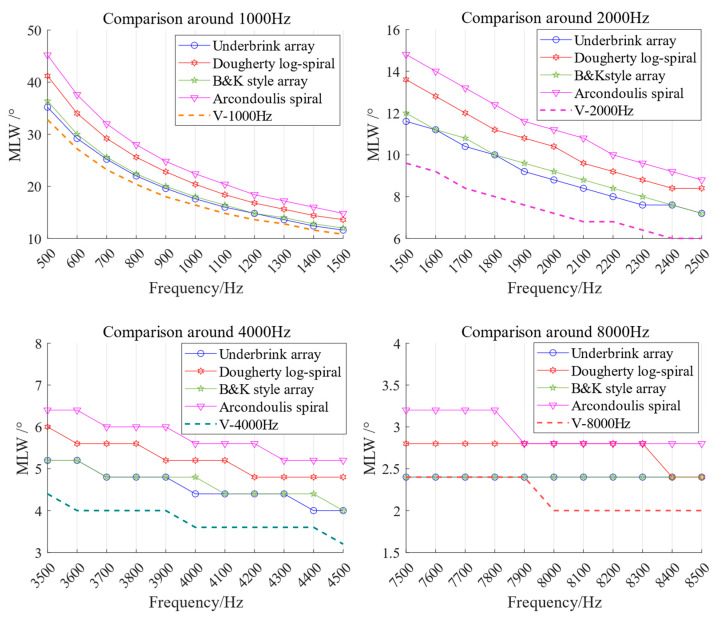
MLW comparison around the target frequency between the V array and the control group arrays, showing that V arrays generally achieve smaller MLW as advantage.

Figure 7 shows the MLW of the V arrays near their target frequencies, in comparisons with the control group arrays. It is shown that MLW decreases with increasing frequency for all the arrays, but eventually stabilizes due to the physical layout of the microphones. The V arrays generally achieve smaller MLW near the target frequencies compared to the control group, indicating better resolution, although the benefit narrows with increasing frequency.

**Figure 8:**
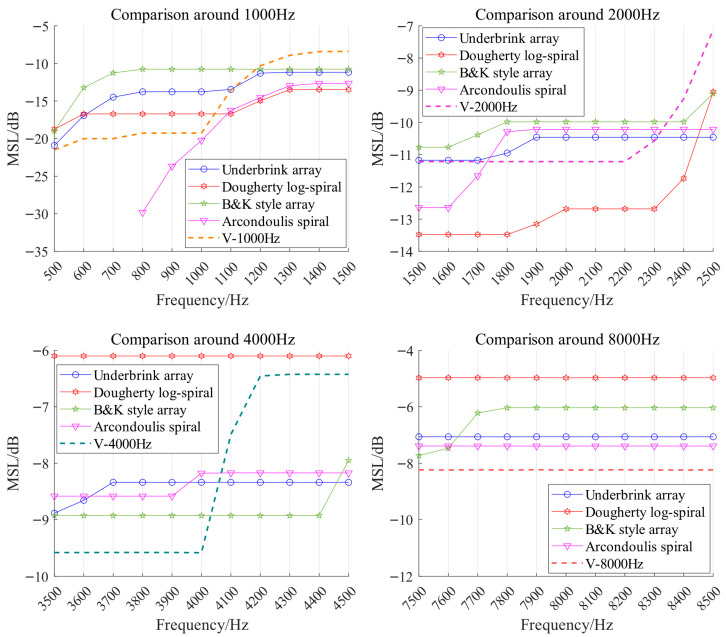
MSL comparison around the target frequency between the V array and the control group arrays showing that V arrays generally achieve lower MSL as advantage.

Figure 8 presents the MSL comparison of all the arrays at the corresponding target frequencies. It is observed that MSL also increases with increasing frequency, but at low frequencies, the PSF’s main lobe can become so broad that sidelobes disappear from the scanning region, making MSL measurement infeasible, as seen with the Arcondoulis spiral array around 800 Hz. Compared to the control group, the V arrays typically achieve lower MSL near the target frequency, though a rapid rise in MSL may occur beyond that point—for example, in the arrays optimized for 1000 Hz and 4000 Hz. The specific MLW and MSL values of each array at the target frequencies are shown in Table 2.

After completing the array performance comparison experiments, additional experiments are conducted to compare the acoustic imaging performance of the arrays using a simulated sound source. In the *X-Y-Z* Cartesian coordinate system, the microphone array was placed on the *X-Y* plane with its center at the origin. A simulated acoustic source is placed at coordinate (0, 0, 1) with a sound pressure level of 60 dB, a tunable frequency and added Gaussian white noise at a signal-to-noise ratio of 10 dB. Acoustic imaging is carried out using the frequency-domain DAS method for both the control group array and the V arrays. The scanning plane spans *X* ∈ [−1, 1] and *Y* ∈ [−1, 1] with a grid step of 0.01 m, located 1 m away from and parallel to the array plane. The imaging dynamic range is set to 12 dB.

**Figure 9:**
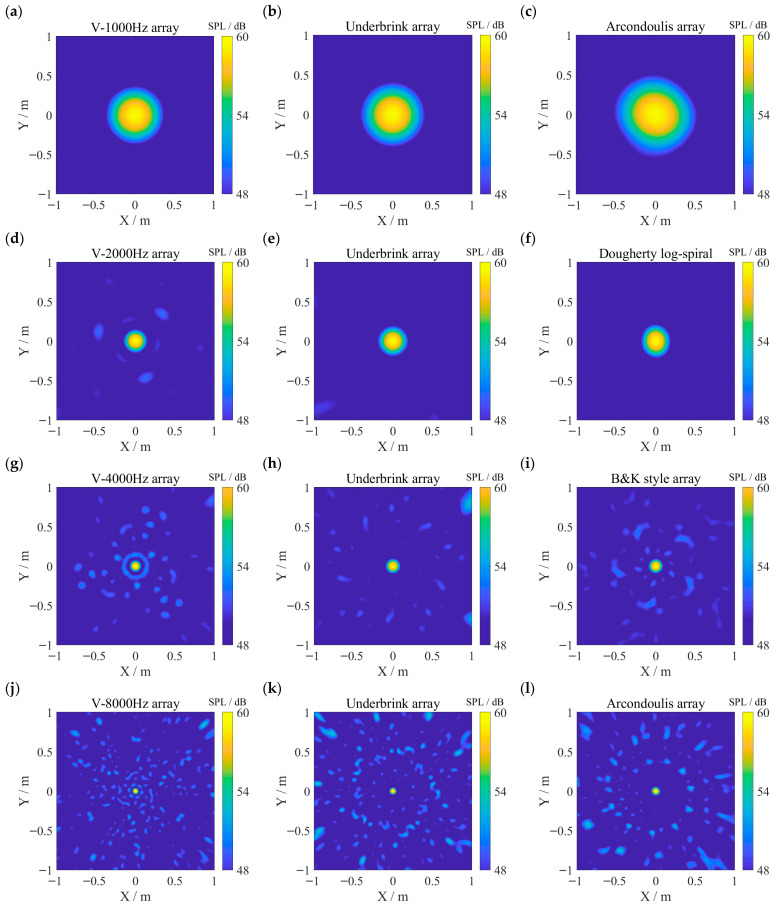
A comparison of frequency-domain DAS imaging results at the target frequencies between the V arrays and those from the control group. The top row (**a**–**c**) presents the imaging results for the 1000 Hz signal. The second, third, and fourth rows show the imaging results for the 2000 Hz, 4000 Hz, and 8000 Hz signals, respectively. The first column (**a**,**d**,**g**,**j**) presents the imaging results from the V arrays. The second column (**b**,**e**,**h**,**k**) presents the results from the control group array that achieves the minimum MLW. The third column (**c**,**f**,**i**,**l**) presents the results from the control group array exhibiting the lowest MSL.

Experiments were conducted with simulated sources at 1000 Hz, 2000 Hz, 4000 Hz, and 8000 Hz. For each frequency, imaging results obtained using the V arrays optimized for that specific frequency and the control group arrays with the best MLW or MSL performance at the same frequency are shown in Figure 9. In the V array results, the source-related main lobe spot appears smaller than in the control group results, consistent with the lower MLW of the V arrays. As frequency increases, the MLW advantage of the V arrays narrows, and so does the difference in main lobe spot size compared to the control arrays. At higher frequencies 4000 Hz and 8000 Hz, more artifacts—spots caused by sidelobes—emerge in the V array results. Notably, at 4000 Hz, the V array exhibits a lower MSL than the Underbrink array, yet shows more imaging artifacts. Given the experimental imaging field corresponds to an elevation angle range of approximately *φ* ∈ [0°, 45°], this suggests that the V array has more sidelobes near main lobe within the imaging region, whereas the highest sidelobe of the Underbrink array lies outside this range. At 8000 Hz, both the V array and the control group arrays produce significant artifacts; however, the artifacts from the V array are less intense, indicating a lower MSL. The presence of increased sidelobe artifacts near the main lobe at higher frequencies in the V array is associated with the lower density of array elements near the array center. Constraining the sidelobe distribution may further improve the performance of the V array, though practical implementation requires additional future work.

**Figure 10:**
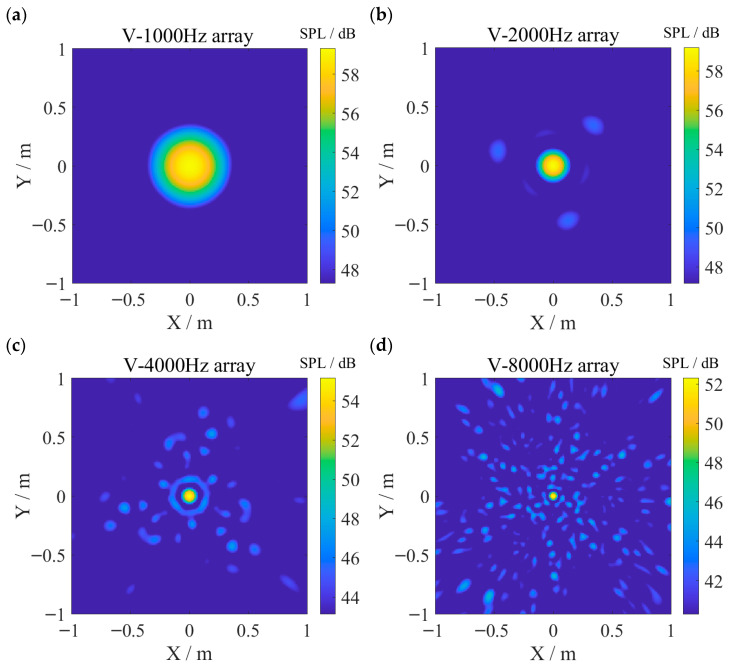
One-third-octave broadband white-noise imaging results of the V array centered at each target frequency, with (**a**–**d**) corresponding to 1000 Hz, 2000 Hz, 4000 Hz, and 8000 Hz, respectively. The imaging results are similar to those of the single-frequency signal, although sidelobe interference becomes more evident with increasing frequency.

In practical industrial scenarios, the exact target frequency is often unknown in advance in acoustic source imaging, and broadband signals are therefore commonly used for imaging. To assess the robustness of the array designed by the proposed method for imaging broadband signals near the target frequencies, the single-frequency simulated sources in the previous experiments were replaced by one-third-octave broadband white noise centered at each target frequency, with a sound pressure level of about 60 dB, and the corresponding V array imaging results are presented in Figure 10. The results indicate that the V array retains stable performance for broadband signals around the target frequency relative to single-frequency cases, with no significant performance degradation and an almost unchanged MLW. As the frequency increases, more sidelobe interference appears compared with the single-frequency imaging results, which is consistent with the previously noted trend of increasing MSL of the V array beyond the target frequency. The performance stability near the target frequency indicates that the proposed method has potential for application in real industrial noise environments.

**Figure 11:**
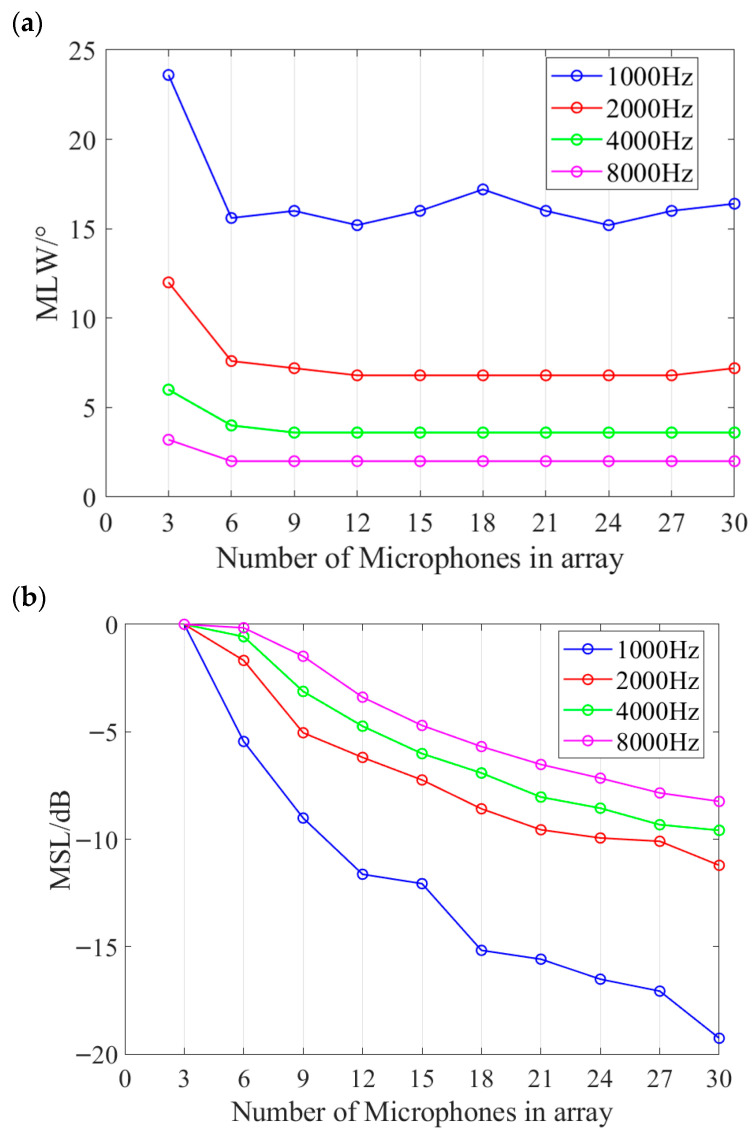
Relationship between the performance of the V arrays for the target-frequency signal and the number of elements during the iterative design process, with (**a**,**b**) corresponding to MLW trend and MSL trend respectively. MLW converges rapidly while MSL gradually decreases.

This work aims to propose a microphone array design method for industrial applications that enables frequency-specific optimization while minimizing the number of array elements. Therefore, it is necessary to investigate how array performance evolves with the number of elements during the iterative design process. The performance of the V arrays optimized for 1000 Hz, 2000 Hz, 4000 Hz, and 8000 Hz during iterative design, as a function of the number of array elements, is shown in Figure 11. The results show that the proposed method prioritizes minimizing the MLW. In all four frequency cases, the MLW approaches its steady value with only six elements and then stabilizes as more elements are added. Higher target frequencies result in more stable MLW trends. In contrast, the MSL continues to decrease steadily with an increasing number of elements and does not show signs of convergence even at 30 elements. Given these trends, users of the proposed method may define performance thresholds for MLW and MSL in practical applications, allowing the iterative process to terminate early once these thresholds are met—thereby avoiding unnecessary microphones and reducing implementation costs.

In this study, the proposed iterative microphone array design method was systematically evaluated through simulation experiment. The results demonstrate that the method significantly enhances acoustic imaging performance with considerable computational efficiency. Compared to classical arrays such as the Underbrink and Dougherty log-spiral arrays, the array designed using the proposed method exhibits a narrower MLW and a lower MSL near the target frequencies. This improvement is attributed to the spatial sampling balance provided by the equilateral triangular subarrays and the precise optimization of element positions via the genetic algorithm. Also, the “additive” iterative strategy circumvents the reliance on a massive initial grid inherent. Consequently, this method substantially reduces the required number of elements while meeting performance specifications, thereby lowering hardware costs. This rapid design capability makes it possible to perform fast array configuration optimization for different machines in factory workshops that exhibit specific frequency characteristics. Experimental results further indicate that although this study primarily optimizes for a single frequency point, the resulting arrays still exhibit good robustness within adjacent frequency bands, demonstrating the method’s potential in real industrial noise environments.

## 5. Future Research Directions

The current research primarily focuses on optimization for single-frequency points. While broadband experimental results indicate that the method can achieve relatively high imaging performance, balancing computational complexity with performance gains for complex broadband signals in real-world industrial scenarios remains a challenge. Future work will explore multi-frequency joint optimization strategies to further enhance imaging quality across the entire frequency band. On the other hand, experimental observations indicate that the sidelobe distribution of the arrays designed by this method tends to degrade at higher frequencies. This is primarily limited by the geometric characteristics of the fixed subarray structure. Future research will attempt to introduce more constraints on subarray placement to improve sidelobe suppression in high-frequency ranges.

## 6. Conclusions

Microphone arrays used in acoustic imaging are limited by their applicable frequency ranges, making it difficult for a single array configuration to meet the detection requirements of diverse targets, like machines and equipment with different characteristic frequencies in the factory workshop. Frequency-specific array optimization is often time-consuming, and the high cost associated with deploying a large number of microphones and their corresponding data acquisition systems further hinders practical applications. To address these challenges, this paper proposes an iterative design method for microphone arrays, in which equilateral triangular subarrays serve as the basic unit. In each iteration, a genetic algorithm searches for the optimal placement of a new subarray to enhance performance at a target frequency. Experiments demonstrate that this method enables rapid array design and allows for controlling the total number of elements via the iteration count, thereby reducing implementation cost.

In performance comparisons with well-known arrays of similar size and element count, the proposed V array typically achieves superior MLW and MSL. Although the performance gap narrows as the target frequency increases, the V array consistently maintains competitive MLW and MSL, with more pronounced advantages at lower frequencies. This work also examines how the V array’s performance evolves during iteration. In general, MLW converges quickly, while MSL decreases gradually as more elements are added. This performance trend allows users to terminate the design process once predefined criteria are met, minimizing the number of microphones required and reducing deployment costs. Meanwhile, imaging experiments using one-third-octave broadband signals centered at the target frequencies demonstrate that the V array maintains good performance for broadband signals, indicating its adaptability to broadband noise in real industrial environments.

Notably, at frequencies of 4000 Hz and above, although the V array maintains an advantage in MSL over the control group, more artifacts caused by sidelobes are observed near the main lobe in the imaging results. This issue is attributed to the sparsity of elements near the array center. Further research is needed to control sidelobe distribution and reduce high-frequency artifacts.

## Figures and Tables

**Table 1:** Selected *ga* function parameters from the Global Optimization Toolbox in MATLAB R2022b.

Parameter Name	Value
PopulationSize	50
EliteCount	2
CrossoverFraction	0.80
MaxGenerations	200
MutationFcn	mutationuniform
CrossoverFcn	crossoverscattered

**Table 2:** MLW and MSL performance of each array at target frequency points.

f/Hz	1000		2000		4000		8000	
Array Type	MLW/°	MSL/dB	MLW/°	MSL/dB	MLW/°	MSL/dB	MLW/°	MSL/dB
V array	16.40	−19.26	7.20	−11.21	3.60	−9.59	2.00	−8.24
Underbrink array	17.60	−13.77	8.80	−10.46	4.40	−8.34	2.40	−7.06
Dougherty log-spiral	20.40	−16.71	10.40	−12.68	5.20	−6.10	2.80	−4.97
B&K style array	18.00	−10.77	9.20	−9.98	4.80	−8.93	2.40	−6.03
Arcondoulis spiral	22.40	−20.23	11.20	−10.21	5.60	−8.18	2.80	−7.39

## Data Availability

We have obtained all the necessary permission.
